# The influence of apical periodontitis on the concentration of inflammatory mediators in peripheral blood plasma and the metagenomic profiling of endodontic infections: Study design and protocol

**DOI:** 10.1016/j.conctc.2020.100686

**Published:** 2020-12-05

**Authors:** A.C. Georgiou, W. Crielaard, P. Ouwerling, W. McLean, D.F. Lappin, S.V. van der Waal

**Affiliations:** a Department of Preventive Dentistry, Academic Centre for Dentistry Amsterdam (ACTA), University of Amsterdam and VU University Amsterdam, Amsterdam, the Netherlands; b Tandheelkundig Centrum Molenvliet, Alphen aan den Rijn, the Netherlands; c Glasgow Dental School, School of Medicine, Dentistry and Nursing, College of Medical, Veterinary and Life Sciences, Glasgow, UK; d Department of Endodontics, Academic Centre for Dentistry Amsterdam (ACTA), University of Amsterdam and VU University Amsterdam, Amsterdam, the Netherlands

**Keywords:** Chronic apical periodontitis, Inflammatory mediators, Endodontic infection, Systemic health

## Abstract

Increased systemic inflammation has been identified in presence of oral disease, specifically endodontic disease. It is important to investigate whether treatment of the oral disease ameliorates systemic inflammation. Furthermore, there is no information about the extent to which different microorganisms may trigger inflammatory response.

**Objectives:**

Primarily (i) to compare the plasma concentrations of inflammatory mediators of apical periodontitis (AP) subjects to controls, (ii) to evaluate whether elimination of the endodontic infection reduces systemic inflammation (iii) to investigate the microbiome of root canal infections. Secondarily i) to correlate the inflammatory mediator data with the microbiome data to investigate whether the type of infection influences the type and severity of the inflammatory condition ii) to examine patterns in the inflammatory mediator data before and after tooth extraction in order to establish a biomarker signature of AP/oral disease.

This is a multi-centre prospective case-control intervention study. The cohort will consist of 30 healthy human volunteers with one or two teeth with a root-tip inflammation and 30 matched healthy controls. Peripheral blood will be drawn at 6 time points, 3 before and 3 after the extraction of the tooth with apical periodontitis. The teeth will be pulverized, DNA extraction and sequencing will be performed.

This study aims to compare the concentration of inflammatory blood plasma proteins in between AP-subjects and controls at different time points before and after the tooth extraction in a systematic and complete way. Additionally the composition of the root canal microbiome in association with the inflammatory response of the host will be assessed.

## List of abbreviations

AP: Apical periodontitis, root-tip inflammation
CBCT: Cone-beam computer tomography
DMFT: Decayed missing and filled teeth
DPSI: Dutch periodontal scoring index
IL-x: Interleukin, a pro- or anti-inflammatory molecule in blood plasma
IMs: Inflammatory mediators, molecules that initiate, sustain or resolve an inflammatory response
MREC: Medical research ethics committee
MSD: Mesoscale discovery platform for inflammatory mediator detection
OTU: Operational taxonomic unit
WMO: Wet medisch-wetenschappelijk onderzoek met mensen

## 1 Introduction

Inflammatory mediators (IMs) can be found in healthy as well as in diseased individuals and is part of the complex biological response of body tissues to harmful stimuli. When looking at reference blood values of healthy, disease-free, individuals it has become clear that even in disease-free humans in peripheral blood, soluble mediators of inflammation can be detected [1]. These inflammatory mediators (IMs) are cytokines, chemokines, acute-phase proteins, soluble adhesion proteins etc. and they are present as a result of homeostasis. Homeostasis is a property of a system in which variables are regulated such that internal conditions remain stable and relatively constant even when in the presence of harmful stimuli. As stated, these ‘healthy’ IMs are the same that can be found in disease and it is when the load of these regulated variables becomes sufficiently great, that the system fails to balance leading to pathophysiology [[2], [3], [4]] In general, the concentration of IMs are lower in health than in disease. This continuous presence of IMs in an apparently healthy individual is also called systemic inflammation or low-grade inflammation. Knowing that disease increases low-grade inflammation, gives us a tool to measure the impact of disease, including oral disease, on systemic health.

Amongst the most common oral diseases is, apical periodontitis (AP) an infection of the root canal system resulting from ingress of micro-organisms from the oral cavity [5]. When the integrity of the surface layers of a tooth crown is lost due to dental caries or trauma, oral micro-organisms gain access to the dental pulp. If the microbial load exceeds the ability of the dental pulp to repair, the dental pulp becomes necrotic and the empty root-canal system becomes populated with micro-organisms. At the root-tip the, now heavy, bacterial load of the root-canal system comes in contact with the periodontal ligament. Microbial products and micro-organisms drive inflammatory changes in this structure leading to recruitment of inflammatory cells and bone resorption which is characteristic of AP [5,6].

A connection between chronic oral infections and the development of adverse systemic health conditions has been historically established [7]). Emerging evidence supports this with new studies proving links between oral bacteria and diseases such as Alzheimer's disease [8]), cardiovascular disease [9] and metabolic syndrome [10]. In the field of endodontics, more specifically, connections between existing illness and apical periodontitis have been made [11,12]. The burden of apical periodontitis on healthy individuals has been also examined. According to a systematic review and meta-analysis we conducted recently, systemically healthy individuals with apical periodontitis presented higher C reactive protein (CRP) values than healthy individuals without AP [13]. These values are related to an increased risk of cardiovascular events [14]. However inconsistencies in study design, treatments and included population that raise questions of the validity of some study results. Additionally, only a couple of IMs where examined in every study, so no complete image of the inflammatory response of the subjects was formed [13].

Studies assessing the association between the presence of selected bacterial species/groups in the apical root canal and expression of mediators in apical periodontitis lesions have been conducted [15,16]. In these studies the different immunologic reactions locally have been associated with differences in root-canal microbiome. To our knowledge, no determination has been made of whether IMs are as a result differentially expressed in peripheral blood.

Therefore, the first purpose of this study is to confirm the current evidence that concentrations of 18 different peripheral systemic IMs in AP subjects differ from controls. The second purpose of this study is to investigate in AP-subjects the course of the inflammatory response, measured in peripheral blood plasma before and after AP treatment with tooth extraction. The third purpose is to map the root canal infection and to correlate its composition with the inflammatory response.

## 2 Objectives

**Primary objectives:** (i) To compare the plasma concentrations of inflammatory mediators of AP subjects to non-AP controls, (ii) to evaluate whether elimination of the endodontic infection reduces systemic inflammation and finally (iii) to investigate the microbiome of root canal infections.

**Secondary objectives:** i) to correlate the inflammatory mediator data with the microbiome data to find out whether the type of infection influences the type and severity of the inflammatory response and resolution thereof, ii) to examine clusters and patterns in the inflammatory mediator data before and after tooth extraction in order to establish a biomarker signature of AP.

## 3 Study design

This is a multi-centre prospective case-control intervention study in which subjects will be followed for 19 weeks.

**Fig. 1:**
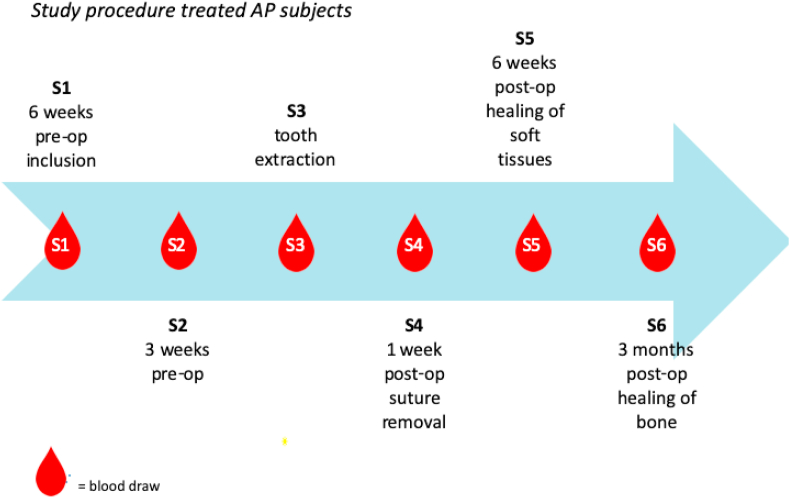
Workflow of the study protocol after the study inclusion.

Thirty subjects with 1 or 2 teeth with apical periodontitis to be treated with tooth extraction will be included. Six peripheral blood samples (S1 – S6; Fig. 1) will be drawn from an antecubital vein in a period from 6 weeks before to 13 weeks after tooth extraction. Sample S3 will be acquired before local anaesthesia is administered, thus before tooth extraction.

Following tooth extraction, the tooth will be collected for microbial analysis. The plasma concentrations of 21 inflammatory mediators will be measured.

Thirty healthy subjects without AP who will be matched for age and sex will serve as controls. From the control subjects blood will be drawn 6 times in 19 weeks. To limit the effect of seasonal fluctuation, blood draw of the matching cases and controls will take place within 14 days.

### 3.1 Study population

#### 3.1.1 Recruitment

The subjects will be recruited from a referral dental practice limited to endodontics, from a referral dental practice limited to implantology as well as from 6 general dental practices.

The control group subjects will also be recruited, from the same practices, that respond to flyers and posters displayed in the waiting rooms with an invitation for healthy volunteers with no dental problems to participate in the study. A database will be created with the information of individuals, that wish to participate. Once a AP case is included a matched individual (for sex and age) will identified from the control database and the individual contacted for inclusion into the study.

#### 3.1.2 Inclusion criteria for AP subjects

In order to be eligible to participate in this study, a subject will have to meet all of the following criteria.

- The subject is 18–80 years old.
- After intra-oral examination, AP should be confirmed with an intra-oral radiograph appearing on the radiograph as a radiolucent area around one or more root tips of the affected tooth. AP will be diagnosed when in the periapical region, the periodontal ligament space is at least twice as wide as in the mid-root regions. A root canal treatment should have a poor prognosis or the patient would rather have the affected tooth extracted.
- No other teeth have AP. To confirm this, teeth will be clinically examined. Discolored teeth or teeth with restorations that will not respond to cold testing or that were tender to percussion or palpation will also be examined with an intra-oral radiograph. In the (pre)molar region, recent bite-wing radiographs will be used to screen for deep restorations or dental caries. When there will be doubts about the vitality of restored or decayed (pre)molars an additional radiograph will be taken. AP will be diagnosed when in the periapical region, the periodontal ligament space is at least twice as wide as in the mid-root regions.
- The subject will complete the medical history questionnaire.
- The subjects volunteer to participate and donate 6 blood samples at 6 different time points and the subject will also have to donate the extracted tooth.
- The subject will not undergo dental hygienist’ treatment during the study.
- The periodontium of the subject is healthy (Dutch Periodontal Scoring Index Category A, DPSI index of maximum 2) [17].

#### 3.1.3 Inclusion criteria for control subjects

In order to be included in the study a control subject will have to meet the following criteria:

- The subject is 18–80 years old.
- No teeth have AP. To confirm this, teeth will be clinically examined. Discolored teeth or teeth with restorations that will not respond to cold testing or that were tender to percussion or palpation will also be examined with an intra-oral radiograph. In the (pre)molar region, recent bite-wing radiographs will be used to screen for deep restorations or dental caries. When there will be doubts about the vitality of restored or decayed (pre)molars an additional radiograph will be taken.
- The subject will complete the medical history questionnaire.
- The subjects volunteer to participate and donate 6 blood samples at 6 different time points.

#### 3.1.4 Exclusion criteria

A potential subject who meets any of the following criteria will be excluded from participation in this study:

- smoking
- pregnancy or lactation
- diabetes mellitus
- chronic inflammatory diseases such as Crohn's disease, hepatitis etc.
- use of antibiotics 1 month prior
- use of corticosteroids or NSAIDs
- chemotherapy or previous head/neck irradiation
- any surgery 6 months prior
- extra-oral swelling pre-operatively
- malaise, colds or influenza
- dental prosthesis carriers with stomatitis
- absence of periapical radiolucency in the presence of tenderness to percussion.
- absence of periapical radiolucency in the absence of a positive sensibility test of the dental pulp
- previous surgery on tooth considered

#### 3.1.5 Late exclusion

Late exclusion can occur when during the following visits:

- the healing is compromised, like alveolitis
- other infection related inflammations occur
- the use of antibiotics for any infection
- the use of corticoids to manage any inflammation.
- dental hygienist visit during the course of the study
- change in health/surgery of any kind
- taking up smoking
- other changes in lifestyle

#### 3.1.6 Sample size calculations

To answer the main question which is to compare cases versus controls a sample size has been calculated with G*Power software. (G*Power 3.1.9.2 software Franz Faul, Universitaet Kiel, Germany) with data retrieved from previous scientific papers where the levels of chosen mediators were determined in serum: for CRP [18]with values 6.6 ± 4.2 mg/L for subjects with AP and 3.9 ± 1.8 mg/L for subjects without AP., for IL-1β [19] with values 225.12 ± 43.72 pg/ml for case and 77.52 ± 31.05 pg/ml for controls and for IL-17A [20] with values 12.48 ± 2.00 pg/ml for cases and 4.43 ± 0.54 pg/ml for controls.

The data will be analyzed statistically utilizing a parametric two-tailed independent *t*-test in comparison of health and disease. The power is set at 80% with an alpha <0.05. Calculated sample sizes were *n* = 17 (CRP), *n* = 17 (IL-1β) and *n* = 15 (IL-17a). These group sizes will also exceed 80% statistical power in the dependent statistical tests, namely the longitudinal data analysis.

In the likely case the data are not normally distributed a non-parametric test (Mann-Whitney U) will be used. Then the sample sizes are calculated at *n* = 22 (CRP), *n* = 22 (IL-1β) and *n* = 20 (IL-17A).

Considering possible no-shows or late exclusion, the group sizes are set at *n* = 30 for the intervention group and *n* = 30 for the controls without AP. The aim was to acquire complete datasets for at least 25 AP plus 25 control subjects.

## 4 Study procedures

**Table 1:** Proceedings in every visit.

visit number	weeks to (−) or from (+) tooth extraction	procedures
S1	−6	- following completion of a medical history form and signing of the informed consent form an intra-oral examination in which the number of decayed, missing and filled teeth (DMFT) will be recorded. Periodontal health will be registered with the Dutch Periodontal Screening Index (DPSI).
		- if recent radiographs are not available, the oral examination will be completed with a radiographic examination with bite-wing radiographs and if necessary additional intra-oral radiographs (in case of deep caries, deep restorations, restoration material in the pulp chamber, tooth discoloration, tenderness to percussion and no response to cold testing).
		- give one 10 mL tube of peripheral blood by antecubital venepuncture

S2	−3	- give an oral update on their medical history
		- give one 10 mL tube of peripheral blood by antecubital venepuncture

S3	0	- give an oral update on their medical history - give one 10 mL tube of peripheral blood by antecubital venepuncture - will have treatment consisting of 1) local anaesthesia 2) Tooth extraction per forceps (if required use of elevator and surgical burr/alveolotomy). Removal of inflammatory tissue from the socket by curretage. 3) Suturing with non-absorbable braided polyester (Ethibond-Excel 4–0, Ethicon, Cincinatti OH, USA), 1–3 sutures. 4) Placement of an aseptic gauze on the wound (patient has to apply pressure for 30 min). - collection and storage of extracted tooth

S4	+1	- will give an update on their medical history
		- will undergo an intra-oral inspection of the extraction wound
		- will have the sutures removed
		- give one 10 mL tube of peripheral blood by antecubital venepuncture

S5	+6	- give an update on their medical history
		- give one 10 mL tube of peripheral blood by antecubital venepuncture

S6	+13	- give an update on their medical history - give one 10 mL tube of peripheral blood by antecubital venepuncture

### 4.1 Blood plasma

Blood will be collected by venipuncture of an antecubital vein using the Vacutainer system (Greiner Bio-One, Alphen aan den Rijn, Netherlands), 9–10 mL in an EDTA plasma tube. Immediately thereafter, the tube with the blood will be centrifuged 10 min × 1000 G and the supernatant plasma (~4 mL) will be taken. Then, the plasma will be cooled to 4 °C and transported to ACTA. There, the plasma will be aliquoted in 0.5-mL aliquots in 1.5-mL microcap centrifuge tubes and stored frozen at −80 °C. Transportation and storage will occur within 24 h of the blood draw.

### 4.2 Inflammatory mediators

Eighteen markers have been selected from our opinion [21] and a Mesoscale Discovery assay (Mesoscale Diagnostics, Rockville MA, USA) will be employed to profile the EDTA-plasma samples.

The selected markers are CRP, IL-8/CXCL-8, MIP-1, MIP-2/CXCL-2, GCSF, IL-1α, IL-1β, TNF- α, IL-4, IL-6, IL-10, IL-12, IL-17A, interferon γ, IFN-γ inducing factor (IGIF), VEGF, RANKL, OPG.

### 4.3 Dental microbiota

Processing the extracted teeth will be according to a previously published protocol [22]. After extraction, the teeth will be stored at −80 °C until cryo-pulverisation. For microbial DNA isolation, the outer surface of the tooth will be wiped off repeatedly with a piece of gauze soaked in 0.5% sodium hypochlorite (NaOCl). The tooth then will be placed in a sodium thiosulfate solution to inactivate the NaOCl. The samples will be cryo-pulverized with the use of a freezer mill (Spex Certiprep, Metuchen, NJ, USA) and the powdered teeth stored frozen at −80 °C.

Samples will be transferred to a sterile screwcap Eppendorf tube with 0.25 mL of lysis buffer (AGOWA mag Mini DNA Isolation Kit; Agowa, Berlin, Germany). Then, 0.3 g of zirconium beads (diameter, 0.1 mm; Biospec Products, Bartlesville, OK, USA) and 0.2 mL of phenol will be added to each sample and the samples homogenised with a Mini-bead beater (Biospec Products) for 2 min. DNA will be extracted with the AGOWA mag Mini DNA Isolation Kit and quantified (Nanodrop ND-1000; NanoDrop Technologies, Montchanin, DE, USA).

For deep microbiome profiling the bacterial composition of each sample will be determined using bar-coded illumina MiSeq sequencing of the hypervariable V4 region of bacterial 16S rDNA. Sequencing data processing will be undertaken using QIIME, sequences will be clustered in operational taxonomic units (OTUs) and assigned to taxonomy. For metagenomic sequencing the extracted genomic DNA will be physically sheared to an average fragment size of 250 bp. The fragmented genomic DNA will be sequenced bi-directionally on a lane of an Illumina HighSeq. From the sequencing data human sequences will be filtered out using BMTagger and functional profiles will be determined using existing software packages (e.g. HUMAnN).

### 4.4 Withdrawal of individual subjects

Subjects can withdraw from the study at any time for any reason if they wish to do so without any consequences. The investigator will withdraw a subject from the study when any of the late exclusion criteria are met.

The recruitment period will continue until the calculated sample size has been reached. If a case-subject drops out, participation of the matched control will be discontinued also. Prior to the start of the study, the control subjects will be informed about this condition.

### 4.5 Follow-up of subjects withdrawn from treatment

Withdrawn subjects will be contacted to determine whether there has been an adverse event or serious adverse event. Subjects who fail to show up for the follow-up session will also be contacted to inquire whether they had forgotten the appointment and to schedule a new appointment.

### 4.6 Study parameters

#### 4.6.1 Primary study parameters

Blood-plasma concentrations of mediators of inflammation.

Composition and function profile of the root canal infection.

#### 4.6.2 Other study parameters

Subject characteristics: signs or symptoms of AP, number of decayed, missing and filled teeth and Dutch periodontal scoring indices, age and sex are used to describe the study population.

### 4.7 Statistical analyses

The data will be entered into SPSS (IBM SPSS Statistics for Windows, Version 25.0. Armonk, NY: IBM Corp) and in STATA. The level of confidence is determined at 95%, α = 0.05.

#### 4.7.1 General

In order to assess the normality of the data, we will visually inspect the Normal Q-Q Plots of the data. To test for normality, we will also use the Shapiro-Wilk test (with p > 0.05 the data will be considered normal) and test for equal homogeneity of variances (Levene's test of variance). In case the data are normally distributed parametric tests will be employed.

When the data are not normally distributed, non-parametric statistical tests will be employed after transformation of the data. The sample size has been determined to be large enough for non-parametric analyses.

#### 4.7.2 Primary objectives

(i) Comparison AP and controls

Parameter means will be compared between AP patients and controls using independent samples Students t-tests.

(ii) Within subject measurements before and after

Linear mixed models with correction for baseline will be performed in order to analyse the longitudinal data. First a crude analysis will be performed, which will be followed by an adjusted analysis for sex and age.

(iii) OTUs are registered and the prevalence of the OTUs will be calculated.

#### 4.7.3 Secondary objectives

Pearson's r is used to correlate between the prevalent OTUs and the concentrations of inflammatory mediators in blood plasma.

Clusters of inflammatory mediators are determined and analysed with K-means analyses with a maximum iteration of 10. The blood plasma samples will be grouped by their presence and concentrations but can also be referenced to the clinical variables. One-way ANOVA will be used to test for differences in expression Z-scores between the clusters.

#### 4.7.4 Missing data

Missing data will not be imputed. Data from drop-out subjects will be used wherever possible. Data from subjects who have withdrawn their consent will be requested if it has already been collected and that data can be used for analysis.

### 4.8 Ethical considerations

#### 4.8.1 Regulation statement

This study will be conducted according to the principles of the Declaration of Helsinki (10th version, October 2013) (www.wma.net) and in accordance with the Medical Research Involving Human Subjects Act (WMO).

#### 4.8.2 Consent

Every subject eligible for participation in the study will be informed about the study by their dentist or the coordinating investigator. If he/she is interested they receive a patient information sheet and the leaflet ‘Medisch-wetenschappelijk onderzoek: Algemene informatie voor de proefpersoon’. Eligible subjects will be instructed to contact the coordinating investigator when they are interested in participation in the study. Subjects give their consent by signing a consent form, but can withdraw from the study at any time and without having to give a reason for withdrawal. Withdrawal the study will not affect the current or future treatments.

#### 4.8.3 Approvals

The study has been approved by the Board of Directors of ACTA.

The study protocol has been approved by the MREC of VU Medical Centre in Amsterdam number 2016/187, NL54832.029.16.

## 5 Discussion

Some IMs that initiate or sustain the inflammatory response are not produced at the site of insult but elsewhere in the body. While being transported via the blood stream to the site of inflammation, it is believed that these IMs may also trigger a response in other cells that are encountered on their journey. On the other hand, IMs that are produced at the site of inflammation may spill into the circulation and thus add to systemic inflammation [1]. At present, associations between oral inflammation and systemic disease have been identified with recent articles finding associations between apical periodontitis (AP) and cardiovascular disease and specifically subclinical atherosclerosis [11,23]. No causal relationships have been established. However, little is known about the impact of AP on healthy individuals.

Some studies have investigated the peripheral blood of subjects and very recently, AP has indeed been associated with elevated concentrations of pro-inflammatory mediators in peripheral blood compared to controls [13]. These IMs are C-reactive protein (CRP), interleukin-6 (IL-6) and asymmetric dimethylarginin (ADMA) which are all pro-inflammatory molecules. In the subjects that were involved in these studies, CRP concentrations were so high that they potentially increase the risk of cardiovascular disease [24,25].

If there is a causal connection: namely that AP causes the increase in systemic IMs, then it is expected to find a decrease in pro-inflammatory mediators after treatment of AP. However, the currently available literature has not revealed such a decline in IMs after treatment of AP (except for complement factor C3 in two studies [26,27]). Great variation in study designs may explain the lack of a unanimous answer to the question of whether AP treatment reduces the systemic inflammatory burden. For instance, in the current literature the treatments of AP vary from tooth extraction, root canal treatment to antibiotics and abscess incisions. Furthermore, great variation in follow-up time points has been reported [13]. This variation in all the studies creates the luck of clarity of whether the treatment had actually removed the infection and consequently the inflammation on histological level is resolved since the effectiveness is questionable. Realistically, the only treatment that leads to a certain resolution of inflammation is the extraction of the involved tooth where the infection is eliminated with removal of the tooth.

Additionally, until now the only way to assess the success of a root-canal treatment and the resolution of inflammation is by radiographic assessment. Due to the need for boney healing to occur to visualize healing, several months have to elapse, sometimes even longer. Radiographic assessment can be achieved either by 2-dimensional (periapical radiograph) or 3-dimensional (Cone Beam CT (CBCT)) means. There are significant statistical differences in studies assessing periapical radiographs and CBCTs, that have implications for periapical diagnosis and for evaluating the outcome of endodontic care [28]. The diagnostic validity, even of 3-dimensional radiographic imaging, especially after endodontic surgery is quite low, with studies showing that up to 42% did not represent actual active inflammatory lesions [29]. Thus there is a need for an alternative diagnostic tool that can determine if inflammation has resolved. It is expected that this study will help towards achieving this goal. Having good certainty that the inflammation is resolved, as the tooth will be extracted, and by examining patterns of IMs in the blood, we can establish blood tests as diagnostic markers for treatment success. In that case the doubt of the radiograph will be eliminated and we would have a simple radiation-free diagnostic tool.

Additionally there is no study, to our knowledge, that has assessed whether there is a connection between the microbiome of the root-canal and the IMs in peripheral blood. With current next-generation sequencing techniques it has become clear that endodontic infections are polymicrobial and that the composition greatly varies even within one root [22,30]. There is yet much to understand of the function of these microbiota in root canal infections and it would be interesting and indeed possibly important to determine which species are involved in the endodontic microbiome and if they can be correlated to certain IMs.

Inflammatory responses are dynamic and responsive to challenges and thus, for establishing an impact of AP on systemic inflammation an intervention type study is to be conducted [21]. Proof that endodontic intervention reduces systemic inflammation in subjects with AP can be provided with a prospective cohort study where subjects are followed in time before and after an intervention. Non-AP subjects should be included as controls. For the resolution of AP, elimination of the infection is mandatory and this can be achieved in two ways: with root-canal treatment or with tooth extraction. Root-canal treatment is not always effective because bacteria can remain due to the complexity of the root canal system [31,32]. Extraction of the infected tooth, however, results in quick and complete resolution of AP [33,34]. Tooth extraction however will elicit an inflammatory response which is triggered by the tissue damage of the procedure. Therefore, the follow-up of individuals should be long enough to be able to measure IMs in the healed situation.

Resolution of inflammation is an active process and not simply a ‘turning off’ of pro-inflammatory pathways. A whole cascade of anti-inflammatory mediators is initiated to clear the site from inflammatory cells. In the case of AP, due to the need for repair of the bone defect in the jaw, we assume that also bone-stimulatory molecules should be produced. In the proposed study, besides evaluating the pro-inflammatory mediators, we also wish to include analyses of anti-inflammatory and bone stimulatory mediators. This will give greater insight into the resolution of AP and possibly a pattern in IMs expression that correlates with AP and the healing of AP [21]. In order to address the research questions about the function of the microbiome and its correlation with systemic inflammation from the extracted teeth, the microbial DNA is obtained and composition will be determined.

In conclusion, with the results of this study, a causal relationship between AP and systemic inflammation can hopefully be established. Moreover, this study will give insight into biomarkers of inflammation which can be used as a future diagnostic tool for the presence of AP and its resolution after root canal treatment.

## Funding

This study is supported by the department of Preventive Dentistry of the Academic Centre of Dentistry Amsterdam (ACTA) and the execution of this protocol will be supported by the annual research grant of the European Society of Endodontology of 2019 (ESE).

## Role of the funding source

The funding sources were not involved in the study design, the collection, analysis and interpretation of data, the writing of this manuscript, or the decision to submit this manuscript for publication.

## Declaration of competing interest

We wish to confirm that there are no known conflicts of interest associated with this publication. The funding sources were not involved in the study design, the collection, analysis and interpretation of data, the writing of this manuscript, or the decision to submit this manuscript for publication.
